# The Role of Iconic Gestures in Speech Comprehension: An Overview of Various Methodologies

**DOI:** 10.3389/fpsyg.2021.634074

**Published:** 2021-04-29

**Authors:** Kendra G. Kandana Arachchige, Isabelle Simoes Loureiro, Wivine Blekic, Mandy Rossignol, Laurent Lefebvre

**Affiliations:** Cognitive Psychology and Neuropsychology, University of Mons, Mons, Belgium

**Keywords:** iconic gestures, speech-gesture integration, methodological considerations, co-network connectivity, multisensory integration

## Abstract

Iconic gesture-speech integration is a relatively recent field of investigation with numerous researchers studying its various aspects. The results obtained are just as diverse. The definition of iconic gestures is often overlooked in the interpretations of results. Furthermore, while most behavioral studies have demonstrated an advantage of bimodal presentation, brain activity studies show a diversity of results regarding the brain regions involved in the processing of this integration. Clinical studies also yield mixed results, some suggesting parallel processing channels, others a unique and integrated channel. This review aims to draw attention to the methodological variations in research on iconic gesture-speech integration and how they impact conclusions regarding the underlying phenomena. It will also attempt to draw together the findings from other relevant research and suggest potential areas for further investigation in order to better understand processes at play during speech integration process.

## Introduction

“Gestures” refer to dynamic movements of the hands (Novack et al., 2016), with “iconic gestures” referring more precisely to manual movements allowing for the transmission of additional or redundant information to the speech they accompany (Kita and Özyürek, 2003; Willems et al., 2007). These gestures greatly contribute to the quality of the information exchange between individuals from an early age onwards. Since the 1990s, numerous attempts have been made to understand the mechanisms underlying the understanding of these gestures and their integration into the associated verbal utterance. Indeed, these gestures appear to possess semantic information that is related to the verbally conveyed message. The notion of “gesture-speech integration” is a central concept in this field. It refers to the implicit cognitive process of combining audio-visual information into a single representation (Green et al., 2009).

To date, studies on gesture-speech integration have employed diverse methodologies, whether in terms of the definition for iconic gestures used, the task or even instructions given to participants. And as suggested by Wolf et al. (2017), the interpretation of verbal and gestural information can be modulated according to the task and/or instruction given to the participants. Our aim is, therefore, to put into perspective the data found in the field of iconic gesture-speech integration by specifically highlighting the methodological variations. Indeed, the diversity of results yielded in this field could be explained by (1) non-identical testing methods (Wolf et al., 2017) (2) overlooking the specificities of iconic gestures or (3) a non-integrative interpretation of results.

First, an integrated and comprehensive definition of iconic gestures will be given to contextualize the focal point of this review. There will then be a focus on the different methodological variations when investigating the links between iconic and verbal information in behavioral, electrophysiological, brain imaging, and brain stimulation studies. Clinical population studies will also be discussed, as they can shed some light on the processes underlying the integration of gestural and verbal information. The discussion will then attempt to integrate all elements to suggest potential avenues for future studies and improve the understanding of the interrelation between iconic gestures and verbal language.

## Characterizing Iconic Gestures: Toward an Integrated and Comprehensive Definition

Iconic gestures convey meaning semantically related to the content of the co-occurring speech (McNeill, 1992). This definition of iconic gestures can be found in the majority, if not all, of the studies conducted on gesture-speech integration. On its own, it might not be sufficient to describe the variety of iconic gestures. These gestures being the central focus of this review, it is proposed to focus on exactly what they represent. The literature identified an iconic gesture as:

(a) A meaningful manual movement (Kita and Özyürek, 2003; Willems et al., 2007);
(b) Temporally aligned to the speech it accompanies (McNeill, 1992; Willems et al., 2007; Habets et al., 2011; Obermeier and Gunter, 2014);
(c) Conveying redundant or complementary information to that present in the co-occurring speech (Krauss et al., 1996; Kita and Özyürek, 2003);
(d) Semi-automatically integrated with speech (Holle and Gunter, 2007; Kelly et al., 2010a);
(e) Providing information on actions (and is then called *kinetograph*), on the shape/size of an object (called *pictograph*), or on spatial relationship between two objects (called *spatial movement*);
(f) Carrying intrinsic meaning but rely on speech to be understood (Krauss et al., 1996; Hadar and Butterworth, 1997; Holle and Gunter, 2007);
(g) consisting of 3 phases (i.e., preparation-stroke-retraction), with the *stroke* carrying most of the semantic content (McNeill, 1992);

Given the variety of iconic gestures, it is essential to know exactly what is being investigated. This will be of a particular interest for this paper. Having these points in mind, the next section will focus on results obtained through various methodologies in behavioral, brain activity and brain stimulation investigations.

## Investigating the Relationship Between Iconic Gestures and Language

Historically, two visions regarding the underlying processes involved in the comprehension and integration of iconic gestures with speech coexist in the literature. On the one hand, Krauss et al. (1991) considered iconic gestures as an epiphenomena of verbal language and do not consider them to have any relevant value in the understanding of the message. On the other hand, most studies and authors now argue in favor of the importance of iconic gestures in language comprehension (McNeill, 1992; Hadar and Butterworth, 1997; Beattie and Shovelton, 2002; Holler and Beattie, 2003; Kelly et al., 2010b), with some considering the gesture-speech integration to be automatic (McNeill, 1992; Kelly et al., 2004).

A recent meta-analysis (Dargue et al., 2019) investigated the effects of co-gesture on speech comprehension. Despite numerous studies showing an enhanced comprehension following the presentation of co-speech gestures, Dargue et al. (2019) highlighted only a moderate beneficial effect. The authors attributed this effect to the diverse methodologies used in the investigation of gesture-speech integration. However, they do not merely consider iconic gestures (the focus of this review) but also other types of co-speech gestures (such as deictic, metaphoric and beat gestures). Subsequently, the authors suggest to investigate the methodological variations within each type of co-speech gesture (Dargue et al., 2019). This review will, therefore, attempt to highlight these methodological aspects among iconic gestures.

First, iconic gesture-speech integration studies can be conducted through behavioral investigations, associated or not with a measure of brain activity or brain stimulation. Second, various experimental designs can be used to assess gesture-speech integration. One way is to modulate the relationship between the iconic gesture and the co-occurring speech. Three types of relationships may be of interest; (a) The information conveyed through iconic gestures may be *redundant* to that conveyed in speech, thereby reinforcing the message. For example, when speaking of a large object, the arm and hand gesture at an increasingly larger amplitude representing the width of the object. (b) Iconic gestures can also be *complementary* and thereby provide additional information to that contained in speech. For example, when speaking of a box one can gesture its shape. (c) The iconic gesture can also contradict the information contained in speech (Dick et al., 2014). In this case, the literature refers to an *incongruency*, most often semantic, between the verbal and gestural information [e.g., gesturing *stirring* while saying *break* (Willems et al., 2007)]. Manipulating the degree of congruency allows to take into account the semantical integration of information present in both modalities (Holle and Gunter, 2007). According to Holle and Gunter (2007), a decrease in performance following the presentation of incongruent information (represented by more incorrect responses or longer reaction times) can be interpreted as a failed attempt to integrate the gestural and verbal information.

Third, the task in itself can modulate the interpretation of results. Some studies require participants to simply observe the stimuli, whereas others require an explicit processing of the information by either focusing their attention on the verbal or gestural information. While observing the stimuli is an ecologically valid approach, focusing on one or the other aspect of the stimuli could seem less natural.

Fourth, investigating different types of iconic gestures could yield different results. As has been mentioned above, iconic gestures can represent actions, manner of movement or physical attributes (McNeill, 1992). More recently, Dargue and Sweller (2018b) also distinguished between typical and atypical iconic gestures.

Finally, other parameters can also be manipulated, such as the type of stimuli presented (i.e., recorded video clips of people gesturing, cartoons, or live presentation of gestures), their content (i.e., presenting single words, sentences, or a narrative), the length of the presented gesture (i.e., the complete gesture or just the stroke), or the visibility of the actor (i.e., if the face is made visible or masked).

The following section will review the literature considering these different parameters.

### Behavioral Investigation of Gesture-Speech Integration

One way to investigate iconic gesture-speech integration is by varying the relationship between the iconic gesture and the co-occurring speech. To the best of the authors’ knowledge, in the gestural domain, Church and Goldin-Meadow (1986) were the first to investigate discrepancies found between produced gestural movements and spoken words. Since then, many authors have contrasted the presentation of congruent vs. incongruent information to investigate the degree of integration between gestural and verbal information (Cassell et al., 1999; Kelly et al., 2004, 2010a; Wu and Coulson, 2005; Wu and Coulson, 2007a,b; Margiotoudi et al., 2014).

All behavioral studies manipulating gesture-speech congruency have highlighted faster reaction times and more correct responses when participants were in presence of congruent pairs compared to incongruent pairs (Kelly et al., 2010a,b; Margiotoudi et al., 2014; Wu and Coulson, 2014; Kandana Arachchige et al., 2018; Zhao et al., 2018; Momsen et al., 2020). These results were found when there was no specific task required (Green et al., 2009; Drijvers and Özyürek, 2018), as well as when participants were required to perform a task where they had to pay attention to the gesture (Kelly et al., 2010b; Margiotoudi et al., 2014; Cohen-Maximov et al., 2015; Nagels et al., 2019; Bohn et al., 2020; Özer and Göksun, 2020b), the speech (Ping et al., 2014; Wu and Coulson, 2014; Drijvers and Özyürek, 2018) or an un-related aspect (Kelly et al., 2010a; Kandana Arachchige et al., 2018; Zhao et al., 2018). Interestingly, a study using a priming paradigm failed to observe a congruency advantage on reaction times when participants were asked to match a target word to a gesture video prime (Wu and Coulson, 2007b). Here, the gesture primes were devoid of any accompanying speech. Since iconic gestures co-occur with a verbal utterance, an essential characteristic is missing for the gestures to be fully considered as *iconic*.

Seeing that the presence of an incongruent iconic gesture appears to hinder performance, whether it is attended to or not, Kelly et al. (2010b) suggested the presence of an obligatory and automatic integration between the two pieces of information. Since then, this automaticity has been put into perspective following data obtained through brain activity investigation. This will be discussed in the following section.

While the investigation of semantic (in)congruency constitutes a big part of the literature, numerous studies have contrasted the unimodal presentation of information (i.e., presenting gesture or speech alone) with a congruent bimodal presentation (i.e., presenting congruent information through both the gestural and verbal modalities) (Beattie and Shovelton, 2001; So et al., 2013; Iani and Bucciarelli, 2017). In a free-recall task, Beattie and Shovelton (2001) and Iani and Bucciarelli (2017) showed an increased information uptake following the presentation of bimodal compared to unimodal information. Yet, using a priming paradigm along with a lexical decision task, So et al. (2013) found no such advantage. It follows that three possible explanations for these contradictory results can be proposed. First, in the latter, it appears that the presented video clips were soundless. As mentioned above, an iconic gesture occurs concurrently to speech. The absence of speech during the video presentation could explain the lack of extra information. Second, participants were asked to respond to a written target. Although the neural correlates involved in the comprehension of spoken and visually presented words appear to overlap (Price et al., 1999), the temporality of the processing involved diverges (Marslen-Wilson, 1984). Third, the type of iconic gestures used in these studies differs. Beattie and Shovelton (2001) and Holler et al. (2009) showed that iconic gestures depicting physical attributes such as relative position, size or shape conveyed more information than other types. More recently, Dargue and Sweller (2018b) distinguished between typical and atypical iconic gestures, the former appearing to be more beneficial to speech comprehension.

The advantage of a congruent bimodal presentation is most noticeable with children. To understand when the ability of integrating gestural with verbal information develops, studies have investigated gesture-speech integration among children. Studies show that by the age of 3, children are capable of integrating iconic gestures representing physical attributes of objects with speech (Stanfield et al., 2013; Macoun and Sweller, 2016; Dargue and Sweller, 2018a; Aussems and Kita, 2019). The ability to integrate action iconic gestures appears to depend on the type of stimuli presentation used. When presenting video clips, Glasser et al. (2018) observed that children from the age of 4 were able to integrate the information from an action iconic gesture with speech to select a corresponding animated clip. Sekine et al. (2015) showed that children from the age of 3 were able to do so when the gestures were presented face-to-face. This real-life presentation advantage has also been observed for adults, particularly for iconic gestures depicting size and position (Holler et al., 2009).

Furthermore, by the age of 5, children presented with live action iconic gestures are able to recall more information compared to when presented with meaningless or no gestures (Kartalkanat and Göksun, 2020). These results were not shown for 3 year olds (Sekine et al., 2015). The age difference between these two studies could here be explained by the nature of the task, children having to pick a picture in the former study (Sekine et al., 2015) and having to produce an explicit answer in the latter (Kartalkanat and Göksun, 2020). In addition, research has shown that from the age of 3, children are able to understand the meaning behind an action iconic gesture in order to open a box in front of them (Bohn et al., 2020). This result was found whether the gesture was presented live or through a video clip. Miyake and Sugimura (2018) observed that the use of directive words (i.e., words indicating in which way an action is carried out) allowed for a better integration of information for 4 year olds. However, the absence of a “Gesture + Speech in the absence of directive words” condition makes it difficult to draw a definitive conclusion. Finally, among iconic gestures, just as for adults (Dargue and Sweller, 2018b), Dargue and Sweller (2020) highlighted that typical iconic gestures benefited comprehension compared to atypical iconic gestures for children.

In contrast to the development of the ability to integrate iconic gesture with speech in children, older adults appear to rely less on gestural information (Cocks et al., 2011). Developing on the suggestion by Thompson and Guzman (Thompson and Guzman, 1999), Cocks et al. (2011) suggested that a weakening of working memory capacities found in aging could explain the difficulty to focus on two different sources of information. More recently, Schubotz et al. (2019) found that older participants, unlike younger ones, did not adapt their words or gestures in a context of shared experience and conveyed less multimodal information when communicating. The results of these studies thus suggest an impairment in the ability to integrate iconic gestures together with speech, which could mirror the capacities developed during childhood (Cocks et al., 2011).

Another population that seems to benefit from a bimodal presentation of information is non-native speakers (Dahl and Ludvigsen, 2014). Dahl and Ludvigsen (2014) and Drijvers et al. (2019) observed an improved understanding of scene descriptions for non-native speakers when they were presented with action iconic gestures depicting physical attributes. By evaluating long-term information retrieval, Kelly et al. (2009) demonstrated that when participants needed to recall words in a foreign language, performances were facilitated when they were exposed to action iconic gestures during the encoding phase (e.g., they found that learning the word *drink* is easier when accompanied by the gesture representing the act of drinking). Other authors have also demonstrated that when presented with degraded verbal information, action iconic gestures improved the comprehension of verbs for non-natives speakers (Drijvers and Özyürek, 2020).

Finally, regarding population, one aspect that has recently started to be taken into account is individual differences. In a recent review, Özer and Göksun (2020a) plead for an assessment of individual differences in the field of gesture comprehension. Indeed, individuals vary regarding their verbal and visual-spatial abilities (Alfred and Kraemer, 2017) and iconic gestures appear to rely on these to be processed (Wu and Coulson, 2014). Given the on-line nature of gesture-speech integration, Wu and Coulson (2014) sought to investigate the involvement of working memory in gesture-speech integration. Using a dual-task paradigm, they showed that visual-spatial, but not verbal, working memory was involved in gesture-speech integration with a higher load on visual-spatial working memory affecting performances on the gesture-speech integration task (Wu and Coulson, 2014; Momsen et al., 2020). An iconic gesture containing semantically related information (McNeill, 1992), the absence of verbal working memory involvement is curious. One potential explanation would consist of not having considered individual differences when loading the verbal working memory span. In fact, the verbal high load condition on the secondary task was completed by having participants remembering 4 numbers (Wu and Coulson, 2014; Momsen et al., 2020). This was the same across all participants, whilst working memory abilities vary across individuals (Jarrold and Towse, 2006). Further research in this field could assess individual differences in a preliminary task and select an appropriate secondary task.

Overall, behavioral studies have highlighted (1) an advantage of congruent bimodal compared to unimodal presentation of information and (2) that iconic gestures seem to be processed in a parallel and automatic fashion with the speech it accompanies. While light variations in individual results can be found, these can be explained by variations in methodological aspects or by not taking individual differences into account.

Beyond the afore-mentioned studies, another large part of the literature has aimed to understand gesture-speech integration within the framework of imaging, electrophysiology and brain stimulation research.

### Investigating Brain Activity During Gesture-Speech Integration

While behavioral studies highlight the interest of adding iconic gestures to speech to enhance observable and quantifiable performance, brain activity can help determine when and where this integration of information takes place. In fact, research in this area is vast. Electrophysiological studies can help reveal the temporal aspects of gesture-speech integration while brain imaging and stimulation studies can shed light on where the integration is taking place. Additionally, just as in behavioral investigations, studies can manipulate the relationship between speech and gesture, use different types of iconic gestures, investigate different populations, etc.

This section will first review electrophysiological studies before focusing on brain imaging and brain stimulation studies.

#### Electrophysiological Studies

As mentioned previously, these studies allow for a temporal approach to semantic integration. More specifically, event-related potentials provide information on the temporal course of the neuronal processes involved following the presentation of a sensory stimulus (Srinivasan, 2005).

Whilst behavioral studies contrasting a congruent and incongruent presentation of information suggested the presence of an automatic integration, electrophysiological studies have highlighted different brain responses depending on whether congruent or incongruent information was presented (Özyürek et al., 2007; Kelly et al., 2010a; Habets et al., 2011).

Studies by Özyürek et al. (2007), Kelly et al. (2010a), and Habets et al. (2011) have demonstrated a larger N400 component following the presentation of incongruent compared to congruent iconic gesture-speech pairs. These studies all investigated action iconic gestures. They did not require the participants to direct their attention to either speech or gesture and presented video clips of the stroke without making the actor’s face visible. According to Holcomb (1993), the N400 component allows to measure the effort required to unify each presented item into an integrated representation. An increase in the N400 component amplitude would, therefore, appear as a complication of this process. Holcomb further suggests that the N400 component reflects a process between the recognition and integration processes (i.e., an activation in a post-semantic memory system). Other authors have suggested that it can reveal a semantic violation in a given context (Luck, 2014), index the level of difficulty to retrieve the associated conceptual representation (Kutas et al., 2006) and arise from a series of processes activating and integrating the target item’s meaning into the presented context (Nieuwland et al., 2020). This component is generally observed in language studies (Kutas and Federmeier, 2011) but can also be elicited by non-linguistic stimuli (Sitnikova et al., 2003).

The presence of a larger N400 component for the incongruent pairs in gesture-speech integration studies (Özyürek et al., 2007; Kelly et al., 2010a; Habets et al., 2011) could thus suggest a difficulty in the semantic processing for these pairs and/or a difficulty in integrating the activated meanings into one unified representation. However, while two studies investigated the incongruency on single words (Habets et al., 2011; Kelly et al., 2010a), the third investigated the incongruency effect within a sentence context (Özyürek et al., 2007). This methodological variation could account for the distinct N400 component site in the three studies. The two studies focusing on single words elicited the largest N400 component in the centro-parietal region, whereas the third study found the largest amplitude in more anterior regions. Using a dual task paradigm, Momsen et al. (2020)’s study also showed the presence of a N400 component being at its largest over anterior channels when presenting sentences. The anterior location is compatible with previous language research eliciting a larger N400 component over anterior regions when in presence of a semantic violation in a sentence context (Hald et al., 2006). This explanation is consistent with the results from another study contrasting ERPs elicited by speech accompanied or not by iconic gestures in a sentence context (Wu and Coulson, 2010). This study showed a larger N400 component over central and centroparietal regions in the absence of iconic gestures (Wu and Coulson, 2010). While the centroparietal effect was found in a sentence context, it was elicited by the absence of an iconic gesture, rather than an incongruent iconic gesture (Momsen et al., 2020). The centroparietal regions, therefore, appear to be involved at a local integration level while anterior regions appear to deal with a global sentence-level integration.

These results led Bernardis et al. (2008) to suggest that the presence of an incongruency slows down the activation of meanings. In line with Thompson and Guzman (1999) and Cocks et al. (2011) proposed that when the presented information was incongruent, the meanings could not be integrated into the working memory, consequently modifying brain activity (Bernardis et al., 2008).

An increase of the N400 component has also been observed when an incongruency was present between a soundless gesture clip and an unrelated word, even when the latter occurred one second after the offset of the gesture clip (Wu and Coulson, 2007b). This result, along with others that highlight the presence of an increased N400 component, despite a long inter-stimulus-interval (Kelly et al., 2004; Wu and Coulson, 2005), appear contradictory to the study by Habets et al. (2011). This research demonstrated that when a gesture and its corresponding utterance were presented 360 ms apart, the incongruency effect reflected by an increased N400 component was not present (Habets et al., 2011). One potential explanation resides in the nature of the stimuli. Wu and Coulson (2007b) presented stimuli that could have been less ambiguous given that in a previous task participants were required to explicitly judge their relatedness to gestures. The stimuli used in Habets et al. (2011)’s study were more ambiguous and hardly understandable without speech.

This incongruency effect was also found in subsequent studies, eliciting a N450 component. This component is thought to be equivalent to the N400 component but specific to a visual/gestural stimulus (Wu and Coulson, 2005, 2007a,b). Just as the N400, it seems to be influenced by the degree of congruency between the iconic gestures and the context in which they are presented (Wu and Coulson, 2005). Indeed, Wu and Coulson (2005) observed an increase of the N450 amplitude when iconic gesture videos (representing either actions or physical attributes of objects) were incongruent to previously presented cartoons. This result was then replicated in the same study when participants were required to relate a target word to the previously presented context (Wu and Coulson, 2005). And in another study assessing the congruency effect between a prime iconic gesture video and target word (Wu and Coulson, 2007b). Interestingly, the N450 component has essentially been demonstrated in studies where the gestures were presented as soundless video clips. This is compatible with the vision of the N450 component as specific to visual stimuli (Wu and Coulson, 2005), as in the absence of speech, the gesture video becomes a visual stimulus.

Furthermore, Holle and Gunter (2007) observed a larger N400 component when an iconic gesture supporting the high frequency homonym or a meaningless gesture followed a low frequency verbal homonym. According to the authors, this suggests that the iconic gesture was able to facilitate the processing of the low frequency homonym. Therefore, by varying the type of gesture presented, Holle and Gunter (2007) demonstrated that iconic gestures can facilitate speech comprehension when the latter needs to be disambiguated. In addition, they questioned the automaticity of gesture-speech integration following the disturbance caused by meaningless grooming gestures (Holle and Gunter, 2007). As attested by these authors, should the integration really be automatic, the presence of grooming movements should not have modified performances. Consistent with this, Kelly et al. (2007) demonstrated that the N400 component to incongruent stimuli could also be modulated by the presence of knowledge on the intentional relationship between gesture and speech. In this case, they found a larger amplitude of the N400 component for incongruent stimuli when participants were aware of the mismatch between the actor uttering the sentence and the one performing the gesture.

Another discrepancy found in the literature concerns early effects. Kelly et al. (2004) reported early sensory effects through a fluctuation of the P1, N1, and P2 components. The P1 component is modulated by selective attention and state of alertness (Luck et al., 2000), the N1 component is influenced by spatial aspects of the stimulus (Mangun, 1995; Hillyard and Anllo-Vento, 1998), and the P2 reflects perceptual processing (Luck and Kappenman, 2011). Kelly et al. (2004) interpreted the presence of these early effects as the creation, through gestures, of a visual-spatial context affecting language processing. According to the authors, the visibility of the actor’s face could have allowed these effects. However, no early effects were found in other studies presenting a visible actor’s face (Wu and Coulson, 2005, 2007a,b). Another explanation could reside in the complexity of the stimuli. In their study, Kelly et al. (2004) repeatedly used the same four simple stimuli (i.e., tall, thin, short, and wide). This repetition could have favored the creation of an expected visual context, thereby eliciting early effects.

Finally, electrophysiological studies have also been conducted on non-native speakers and, recently, children. For non-native speakers, Drijvers and Özyürek (2018) observed a larger N400 component for incongruent stimuli pairs. This effect disappeared in the event of degraded speech for non-natives, but remained for native speakers (Drijvers and Özyürek, 2018). The authors theorized that a minimum quality of the auditory stimulus is required for the integration process to take place for non-native listeners (Drijvers and Özyürek, 2018). A subsequent study corroborated these results, revealing that unlike for native speakers, non-native speakers do not benefit from visible speech (i.e., visible phonological information) in a degraded auditory context (Drijvers and Özyürek, 2020).

To the best of the authors’ knowledge, only one electrophysiological study investigating gesture-speech integration in children has been conducted. In their study, Sekine et al. (2020) observed a larger N400 component for the incongruent trials compared to congruent ones. In line with data from behavioral studies on the development of gesture-speech integration in children (Stanfield et al., 2013; Sekine et al., 2015; Glasser et al., 2018), this study suggests that by the age of 6, children possess a qualitatively similar processing of gesture-speech information to adults.

In conclusion, although a late semantic effect has consistently been elicited, the same cannot be said for early effects. Other than for non-native speakers, results plead in favor of the existence of a semantic link between the iconic gestures and co-occurring verbal utterance. Electrophysiological studies thus corroborate results from behavioral studies. The absence of consistent results relating to early effects could be explained by the type of iconic gesture presented. As highlighted, iconic gestures comprise a variety of more or less complex gestural movements and can be redundant or complementary to speech. Moreover, the presence of late semantic effects is not exclusive to the presentation of iconic gestures. Consequently, although this constitutes a good first step in understanding the neural process involved in iconic gesture-speech integration, further investigation is required to deepen an understanding of this research area.

#### Brain Imaging and Brain Stimulation Studies

One way to enhance our understanding of the neural processes involved in gesture-speech integration is by using functional Magnetic Resonance Imaging (fMRI). This would allow to highlight which brain regions are involved in the processing of iconic gestures and understand their relationship to speech.

Most of the fMRI studies have been conducted with simple observation tasks (Willems et al., 2007; Dick et al., 2009, 2012, 2014; Green et al., 2009; Holle et al., 2010; Straube et al., 2011; Demir-Lira et al., 2018). Wolf et al. (2017) justified this choice by highlighting the possible motor-related artifacts caused by participants having to produce a motor response. In fact, Willems et al. (2007) observed an involvement of typical motor areas (such as the premotor cortex) in language processing, and typical language areas (such as Broca’s area) in action processing. A motor involvement has also been suggested by behavioral studies (Ping et al., 2014; Iani and Bucciarelli, 2017). These studies showed that hand/arm movements produced by the participants hindered their ability to integrate gesture-speech information. Interestingly, this interference effect was not observed when participants were required to move their foot/leg (Ping et al., 2014; Iani and Bucciarelli, 2017).

With regard to gesture-speech integration, three main regions were found to be involved: the left inferior frontal gyrus (left IFG), the middle temporal gyrus (MTG) and the posterior superior temporal sulcus (pSTS).

An increase in the activity of the left IFG was observed during the presentation of iconic gestures (Dick et al., 2009) when they were incongruent to speech (Willems et al., 2007, 2009) and when they conveyed complementarity (Holler et al., 2015) compared to redundant information (Dick et al., 2014). But this enhanced activity was not found when comparing the presence and absence of iconic gestures (i.e., comparing a Gesture + Speech condition to a Gesture + Unrelated Movement or to a Speech Alone condition) (Holle et al., 2008; Dick et al., 2009; Green et al., 2009; Straube et al., 2011). The involvement of the left IFG in gesture-speech integration, therefore, appears to not merely be restricted to combining information, but rather to detect incompatibilities (Willems et al., 2007, 2009) and/or create a new coherent representation from two ambiguous inputs (Dick et al., 2014; Holler et al., 2015). This is consistent with viewing the left IFG as a unification site (Zhu et al., 2012). The process of unification allows for either lexically retrieved information, or meanings extracted from non-linguistic modalities to be integrated into one representation (Hagoort et al., 2009). Studies in the language domain suggest a functional separation between anterior and posterior regions of the IFG, the former being linked to controlled semantic retrieval and the latter to general selection processes (Gough et al., 2005; Humphries et al., 2007; Lau et al., 2008; Hagoort et al., 2009). Consequently, one possibility would be to allocate the integration of complementary information to anterior regions and the processing of incongruency to the posterior regions.

The role of the IFG as a unification rather than an integration site could explain why its activation is not limited to iconic gestures. Indeed, Willems et al. (2009) highlighted an increase of the left IFG activation following the presentation of incongruent pantomimes. Straube et al. (2011) observed an increased activation for congruent metaphorical but not iconic gestures. The explanation would here reside in the higher effort needed to comprehend metaphorical gestures as they represent abstract concepts.

Therefore, rather than being exclusive to iconic gesture processing, the left IFG is involved when (1) a deeper processing of information is required, (2) and a new representation of the information must be created and/or (3) when there is an incompatibility between several representations that needs to be resolved. In a recent study investigating the effects of transcranial magnetic stimulation (TMS), Zhao et al. (2018) caused slower reaction times on a gesture-speech integration task after stimulating (and therefore disrupting) the left IFG and left pMTG. TMS is a non-invasive neuro-stimulation technique that disrupts neuronal activity by inducing a virtual lesion (Pascual-Leone et al., 2000). This highlights the cortical areas involved in a task and the temporality at which this contribution takes place (Hallett, 2000) and demonstrates a causal relationship between a neural process and the behavior observed on the task (Rossini and Rossi, 2007). Hence, results from Zhao et al. (2018) suggest a reduction in the integration of iconic gestural and verbal information following a disruption of the left IFG and pMTG. Because these effects were obtained through two different protocols, the authors suggested that the IFG and pMTG contribute to gesture-speech integration, respectively, to retrieve contextual semantic information and stored semantic information (Zhao et al., 2018).

An involvement of the MTG in gesture processing has also been put forward by several studies (Dick et al., 2009, 2014; Willems et al., 2009; Holler et al., 2015; Demir-Lira et al., 2018). Still, just as for the IFG, results vary depending on the nature of the stimuli. Dick et al. (2009) observed an increase in bilateral MTG activity in the presence of gestures though it did not discriminate between co-speech gestures and meaningless gestures. A specific activity increase was highlighted by Willems et al. (2009) for incongruent pantomimes (i.e., gestures that can be understood in the absence of any speech) but not for incongruent iconic gestures. However, when investigating the effects of complementary iconic gestures (rather than redundant), several studies have demonstrated an increased MTG activation (Dick et al., 2014; Holler et al., 2015; Demir-Lira et al., 2018). While Dick et al. (2014) highlighted this increased activity on the left MTG for adults, Demir-Lira et al. (2018) observed it on the right MTG for children. The difference of location has been suggested to reflect the possible use of additional cues in children compared to adults (Demir-Lira et al., 2018). Finally, Holler et al. (2015) observed that when listeners were specifically addressed, the presence of iconic complementary gestures elicited an increased right MTG activation.

Wagner et al. (2001) suggested that the left MTG could work together with the left IFG to retrieve semantic information (Kuperberg et al., 2008). Although the IFG appears to be sensitive to congruency (Willems et al., 2009), the MTG does not. In the language domain, Badre et al. (2005) suggested that the MTG was sensitive to target association but not competition. This is consistent with an involvement of the MTG in integrating complementary iconic information with speech.

The third main site that appears to be involved in gesture-speech integration is the left pSTS (Holle et al., 2008; Straube et al., 2011; Demir-Lira et al., 2018). In the field of recognition, this region appears to be involved in the integration of multimodal information (Beauchamp et al., 2004a,b). In language comprehension, the STS is activated during speech presentation (Crinion et al., 2003) with the left temporal cortex critically involved in the storage and retrieval of linguistic information (Hagoort, 2013). In the gesture-speech integration domain, studies have again yielded mixed results. In some studies, although an increased activation of the left STS was found in the “Speech + Gestures” condition, this activation either wasn’t sensitive to the meaning of gestures (Willems et al., 2007; Dick et al., 2009, 2012, 2014), or was greater in the case of incongruent pantomimes but not iconic gestures (Willems et al., 2009). These latter results, along with the observed activation of MTG for pantomimes, led Willems et al. (2009) to suggest that pSTS/MTG was involved in the integration of information on a relatively stable conceptual representation. According to the authors, the nature of co-speech gestures (i.e., language-dependent) require that they be integrated at a higher level, given that they involve the creation of a novel representation.

Interestingly, this very explanation was later taken up by Straube et al. (2011) to explain the presence of a greater left pSTG (posterior superior temporal gyrus) activity in the “Speech + Iconic” and “Speech + Metaphoric gestures” conditions compared to Speech Alone. Though these authors offer the same role of pSTS/pSTG, they seem to disagree on which co-speech gesture it processes. Other studies have shown an involvement of the left pSTS in iconic gesture processing. Comparing the presence of iconic gestures to grooming movements, Holle et al. (2008) highlighted a greater activation of the left pSTS for the former. A different study replicated these findings by observing a bimodal enhancement over the pSTS/STG region when in presence of “Speech + Iconic gestures” (Holle et al., 2010). It also observed that this augmentation was greater in the context of degraded speech (Holle et al., 2010). Similarly, a previous study showed an increased activation of left superior temporal areas when the presented speech mismatched the sentence context (Willems et al., 2007). Holle et al. (2010) purported the existence of a sensitivity gradient within the pSTS/STG. This, with anterior portions being sensitive to speech processing and posterior regions (near the temporo-occipital, TO, junction) being sensitive to gestural information (Holle et al., 2010). This is consistent with a study by Green et al. (2009) that demonstrated an augmented activation at the left TO junction in the presence of Familiar Speech + Iconic gestures.

Brain imaging studies investigating gesture-speech integration in children are rare. When comparing the presence of iconic gestures, metaphoric gestures and grooming movements, Dick et al. (2012) observed an enhanced left pSTS activation for all types of movements relative to a baseline fixation activity. More recently, Demir-Lira et al. (2018) highlighted an increased left pSTS activity for complementary iconic gestures compared to redundant or no gestures. Because Dick et al. (2012) have not detailed the type of iconic gesture used or the relationship between the iconic gestures and speech (i.e., whether they were redundant or complementary), a direct and definitive comparison would be speculative. Furthermore, it is possible that limiting their sample to 9 children did not allow to investigate precise activation differences. Another possible explanation resides in the presence of methodological dissimilarities (Holle et al., 2008). More precisely, as the authors have highlighted, the relationship between gesture and speech as well as their level of integration could be key. It is possible that the pSTS serves as a local integration site [i.e., when the gesture is required to be integrated with the verbal unit (Holle et al., 2008)], and the IFG would act as a global integration site (i.e., where integration is required on a sentence level) (Willems et al., 2007; Dick et al., 2009; Holle et al., 2008, 2010). This supports the presence of a pSTS activation for complementary iconic gestures, the integration taking place on a local unit level.

Two main conclusions can be drawn from these findings. First, given the methodological variations (such as tasks, type of gesture or relationship between gesture and speech), defining one precise neural network involved in iconic gestures/speech comprehension is laborious. Yet, this variation can be beneficial for a more precise understanding of what is involved when, during iconic gesture-speech integration. Second, because these three areas (i.e., IFG, pSTS, and MTG) appear to be involved in various degrees and at different moments, connectivity studies could shed some light on the matter.

Hein and Knight (2008) suggested that the function of STS varies according to the nature of the co-activated network. This vision supports the idea that the same brain region can result in different cognitive processes depending on the nature of the task or stimuli involved. The existence of a task-dependent co-activated network reconciles the numerous observations mentioned hereinabove.

Recent studies have investigated the connectivity signature of co-speech gesture integration (Straube et al., 2018) and the spatial-temporal dynamics of gesture-speech integration (He et al., 2018). While their results support the key role of pSTS (He et al., 2018; Straube et al., 2018) and IFG (He et al., 2018) in gesture-speech integration, the gestures they investigated “could be comprehended even without accompanying speech” (Straube et al., 2018). Therefore, this does not fit the criteria to be classified as *iconic* gestures. Future research could attempt to explore the connectivity signature of iconic gestures integration.

Similarly, Drijvers et al. (2018) investigated the spatiotemporal changes in cerebral oscillations when the presence of gestures enhances clear or degraded speech. The study of brain oscillations has regained interest in the last decade (Wang, 2003; Ward, 2003; Weiss and Mueller, 2012; Başar, 2013) as it can provide complementary data to those obtained via fMRI on how brain activity relates to cognitive performances (Ward, 2003). A suppression of alpha and beta activity is found in regions that are engaged in a task (Jensen and Mazaheri, 2010; Quandt et al., 2012), while an increase in gamma activity is linked to an enhanced cognitive activity (Fitzgibbon et al., 2004; Jensen et al., 2007). Previous research has shown differentiated alpha and beta rhythms whether the gesture observed was iconic or deictic. This is consistent with alpha and beta rhythms being closely linked to the allocation of visual-spatial attention (Quandt et al., 2012) and that iconic and deictic gestures are processed differently. When gestures enhanced communication in a degraded speech context, Drijvers et al. (2018) demonstrated a greater suppression of alpha and beta activity over motor regions (hand motor area and supplementary motor area). According to the authors, this could suggest an attempt of imagining the action to aid comprehension (Drijvers et al., 2018). This is in agreement with a previous study showing alpha and beta power suppression in the precentral gyrus regions during motor imagery (De Lange et al., 2008). An alpha and beta suppression in frontal regions (Momsen et al., 2021) and more specifically in the left IFG and left pSTS/MTG, STG regions (Drijvers et al., 2018) is consistent with their role in gesture-speech integration highlighted by imaging studies. An increase in gamma power in the left temporal lobe was found at the presentation of the gesture’s stroke and co-occurring speech, suggesting an attempt to integrate both information (Drijvers et al., 2018).

Overall, results in brain activity studies show the importance of knowing exactly what type of gesture is involved and its relationship to language. We have underlined that these two variables, along with the task involved, can modulate the interpretation given to the results and could explain apparent discrepancies between studies. In electrophysiological studies, the complexity of the presented task and iconic gesture can influence whether or not early sensory components are modulated. Mismatch paradigms consistently elicited the presence of a late semantic component. Yet, this component varies in its timing (N400–N450). We suggest that this variation was due to the stimuli that were used in the tasks (e.g., soundless video clips, audio-visual gestures). Brain imaging studies variously showed an involvement of the left IFG, left pSTS and MTG in gesture-speech integration. These activations appear to mainly depend on the nature of the relationship between iconic gesture and speech (i.e., redundant, complementary, or incongruent), as well as on the task. These variations plead in favor of the existence of a task-dependent co-activated network.

### Investigating Gesture-Speech Integration in Clinical Populations

The study of behavior and cognition of clinical population allows for a better understanding of healthy cognition (Eysenck, 2014). The presence of an impairment of gesture-speech integration in patients could thus improve the understanding of the processes underlying the same integration in neurologically intact individuals. However, seemingly inconsistent results have also been highlighted within the same clinical population. This section will attempt to reconcile these apparent discrepancies by focusing on four clinical groups: aphasia, specific language impairment, autism spectrum disorder and schizophrenia.

Aphasia is an acquired disorder that can affect both language production and comprehension (Preisig et al., 2018). While language and gesture production have been vastly studied, literature on gesture comprehension is quite sparse.

In 1972, Gainotti and Ibba observed an impairment of pantomime comprehension among aphasic patients. This result was later replicated for aphasic patients presenting mono-hemispheric cerebral lesions compared to healthy participants and non-aphasic brain damaged patients (Gainotti and Lemmo, 1976). While these were among the first studies to focus on gesture comprehension in aphasia, they do not investigate iconic gestures, nor specify the type of aphasia involved. A couple of years later, a new study investigating pantomime processing showed that performances depended on the type of aphasia (Ferro et al., 1980). Ferro et al. (1980) showed that patients with Global, Wernicke and Transcortical aphasia presented lower performances at the Gesture Recognition task, compared to patients with Broca, Anomic or Conduction aphasia. The authors associated these results with the presence of lesions in the left posterior regions, involved in gesture identification. Since then, to the best of the authors’ knowledge, studies have not differentiated their results according to aphasia type.

More recently, several studies investigated co-speech gesture-speech integration in aphasia (Eggenberger et al., 2016; Cocks et al., 2018; Preisig et al., 2018). Preisig et al. (2018) showed that during live conversations, co-speech gestures (of all types) attracted the attention of aphasic patients and were more fixated than abstract gestures. The authors suggested that these patients may benefit from the bimodal information presentation to compensate a verbal deficit (Preisig et al., 2018). However, because no task was involved, it is unclear whether patients understood and processed the meaning of these gestures. Another study required patients to explicitly integrate the iconic gesture meaning with the co-occurring speech by deciding whether they were congruent or not (Eggenberger et al., 2016). Results showed that patients performed better when presented with congruent compared to incongruent pairs or associated with meaningless movements. Eggenberger et al. (2016), therefore, concluded that congruent iconic gestures could enhance comprehension for patients with aphasia. Cocks et al. (2018) moderated this claim by observing poorer performances in patients when they were asked to integrate speech with complementary iconic gestures. Although these studies did not distinguish performances according to aphasia type, they do confirm the need for a precise qualification of the type of iconic gesture and its relationship to speech. Indeed, it appears that the advantage of a bimodal presentation is only present in the case of redundant and not complementary gestures. Eggenberger et al. (2016) have also proposed that future studies take individual differences into account, particularly when studying clinical populations.

Another pathology presenting a heterogeneous profile of language deficits, and particularly a limited verbal comprehension, is Specific Language Impairment (SLI) (Evans and Brown, 2016). Because this disorder is characterized by the presence of a language impairment in the absence of non-verbal cognitive impairments (Botting et al., 2010), it is of particular interest for the investigation of co-speech gesture integration. Using a Speech/Gesture Integration task [a paradigm created by Cocks et al. (2009)], Botting et al. (2010) not only highlighted poorer performances for SLI children, but also showed that they made more gesture-based errors. This would suggest that these children, although they did recognize hand movements, were unable to either extract the meaning from the gestures, or integrate it with the sentence context (Botting et al., 2010). These findings were later replicated by Wray et al. (2016), even after controlling for non-verbal cognition abilities. The difficulty for SLI children to integrate gesture meaning into a sentence context is consistent with language studies showing difficulties in integrating contextual information (Botting and Adams, 2005; Ryder et al., 2008). However, using a different paradigm, a study by Perrault et al. (2019) showed better performances when children with language disorders were faced with iconic gestures compared to typically developing (TD) children and children with autism spectrum disorders (ASD). Interestingly, the gestures in this study were devoid of sound and did not require any form or contextual integration to be understood; co-speech gestures used in Botting and Adams (2005) and Wray et al. (2016) studies were complementary to speech, while Perrault et al. (2019) used gestures *in place of* speech. More recently, Vogt and Kauschke (2017) highlighted a beneficial effect of bimodal iconic gesture presentation on word learning for SLI compared to TD children. These apparent contradictory results can be explained by the presentation format of the stimuli. Vogt and Kauschke (2017) presented face-to-face gestures while the previously discussed studies with null effects presented video clips (Botting et al., 2010; Wray et al., 2016). As has already been highlighted in this review, real-life gesture presentation has shown to be more efficient in improving comprehension (Holler et al., 2009; Sekine et al., 2015).

Perrault et al. (2019) also investigated gesture comprehension among ASD children. These children performed worse than TD and SLI children for co-speech gestures. The results were partly comparable to those of Dimitrova et al. (2017). These authors showed that compared to complementary co-speech gestures (for which performances were indeed poorer), redundant gestures improved performances for ASD children. They also demonstrated that gesture comprehension was linked to receptive language abilities.

Finally, several studies focused on the perception of gestures in patients with schizophrenia. An older study has shown a general impairment of gesture recognition (Berndl et al., 1986). However, there are numerous types of gestures, none of which are entirely processed in the same manner. In fact, patients present an inability to understand the meanings being metaphors or abstract concepts (Kircher et al., 2007). This is consistent with recent studies suggesting that recognition of metaphorical compared to iconic gestures is selectively impaired (Straube et al., 2013, 2014; Nagels et al., 2019). Although iconic gesture recognition appears to be preserved, studies investigating the neural processes involved yield some interesting results (Straube et al., 2013, 2014; Schülke and Straube, 2019). Straube et al. (2013) found a disturbance in the activation of the left pMTG/STS and IFG for metaphorical gestures. A subsequent study specified the existence of a negative correlation between positive symptoms of schizophrenia and connectivity between the left IFG and left STS (Straube et al., 2014). In contrast, the activation of the left STS (and its connectivity to the left IFG and left MTG) for iconic gestures was comparable to that of healthy participants (Straube et al., 2014). Using tDCS, a recent study showed improved performances on a semantic-relatedness task when stimulating the frontal and fronto-parietal regions (Schülke and Straube, 2019). In schizophrenia, it would, therefore, appear that gesture recognition is selectively impaired for metaphorical gestures and preserved for iconic gestures. However, all these studies presented redundant iconic gestures. Given (1) the apparent distinction in processing redundant vs. complementary information and (2) the processing similarities for metaphorical and complementary iconic gestures (both involving IFG unification processes), contrasting these two types of gestures could be an avenue for further research.

In summary, although data from clinical studies presents a somewhat confusing picture, this can be resolved by considering the relationship between iconic gestures and the co-occurring speech as well as individual differences. Results for aphasic patients indeed suggest a differentiated effect of iconic gestures on comprehension, with redundant iconic gestures improving it and complementary iconic gestures hindering it. Studies on SLI reveal a positive effect of iconic gestures on comprehension, but only when these are presented face-to-face. A clear distinction of performance between complementary and redundant iconic gestures is found with ASD children as only the presence of the latter enhances comprehension. Finally, iconic gesture comprehension seems to be preserved in schizophrenia. Having said that, existing studies have predominantly focused on investigating redundant iconic gestures. Exploring the comprehension of complementary iconic gestures would consequently allow to paint a more complete picture of gesture-speech comprehension in schizophrenia.

## Conclusion

Following this overview on the investigations of gesture-speech integration and the role of iconic gestures in language comprehension, an undeniable observation is the diversity of methods used, and the associated variation in results. Studies investigating neurologically intact individuals agree on attributing an active role to iconic gestures in improving language comprehension, particularly in an unfavorable listening context. But this does not imply that the gesture-speech integration is carried out in an automatic fashion and/or stems from a “unique integrated system” (McNeill, 1992; Kelly et al., 2010b). Rather, behavioral, electrophysiological and clinical studies results appear to plead in favor of the existence of two distinct systems, one being able to compensate the other in the event of an impairment (Perrault et al., 2019). Behavioral results suggest that gesture-speech integration can be modulated by the semantic overlap between both modalities (Holle and Gunter, 2007), intentionality (Kelly et al., 2007), or more generally in the presence of situational factors (Holle and Gunter, 2007). In other words, the automaticity of gesture-speech integration can be affected when attention is explicitly directed toward integration or when the task requires a controlled cognitive process, such as a lexical or semantic decision (Kelly et al., 2010b).

In clinical studies, depending on the pathology, the authors have identified either a parallel impairment of the verbal and gestural channels (thereby supporting the “unique integrated system”), or a preservation of the gestural channel allowing for better performances when the verbal channel is impaired. Discrepancies can also be found within the same pathology, depending on the relationship between gesture and speech and the task involved. In aphasia and ASD, the automaticity of integration is consistent with the poorer performances in presence of complementary iconic gestures but undermined in presence of redundant gestures. In SLI, the essential variable seems to be the presentation format of the stimuli. Further research on gesture-speech integration in schizophrenia would be needed in order to be able to distinguish between performances depending on the type of iconic gestures involved. All things considered, this variation in methodology could thus explain the current discussion regarding the automatic nature of gesture-speech integration.

These methodological variations could also serve as framework to analyze brain activity data. This review has emphasized the necessity of future research investigating the co-activated neural networks underlying gesture-speech integration for various types of iconic gestures, and differentiating the redundant from the complementary iconic gestures. Relatively new to the field, TMS (as well as its combination with neuroimaging or electrophysiological techniques) could also offer an interesting perspective on areas involved in gesture-speech integration. The combination of techniques would allow for a more precise qualification of the role and timing of the different regions involved. This is particularly relevant in relation to exploring the co-activated neural network involved in the processing of iconic gestures integration, as has been highlighted in this article. TMS studies could also help to shed some light on the presence or absence of early effects observed in electrophysiological studies. However, as mentioned previously, special attention must be paid to precisely characterizing the type of iconic gesture used as well as its relationship to speech (i.e., whether it is redundant, complementary, or congruent).

Finally, studying the cognitive processes underlying gesture-speech integration could also enhance our understanding of the latter. However, as has been emphasized, merely investigating cognitive processes does not imply taking individual variability into account. Therefore, considering individual differences is essential for a correct understanding of what is involved in gesture-speech integration.

Following this overview, Table 1 provides a non-comprehensive but extended summary illustrating the possible variables that can be manipulated in the investigation of gesture-speech integration with an example of a study for each element. Appendix A presents a detailed summary of these variables for the studies explored in this paper.

**TABLE 1:** Types of possible investigation and possible dependent variables in the study of iconic gestures in comprehension.

Methodological aspects	Possible variations
Investigation	– Behavioral (Beattie and Shovelton, 2002). – Electrophysiology (Wu and Coulson, 2007a). – Transcranial magnetic stimulation (Zhao et al., 2018). – Transcranial direct current stimulation (Cohen-Maximov et al., 2015). – Functional magnetic resonance imaging (Holle et al., 2008). – Magneto encephalogram (Drijvers et al., 2018). – Eye tracking (Beattie et al., 2010).
Gesture-speech integration	– Implicit (Sekine et al., 2015) or Explicit (Perrault et al., 2019).
Task during stimuli presentation	– Passive observation (Habets et al., 2011). – Dual task paradigm (Wu and Coulson, 2014). – Lexical decision (So et al., 2013). – Attentional task (Green et al., 2009). – Target relatedness task (Ping et al., 2014). – Stroop-like task (Kelly et al., 2010a).
Attention during stimuli presentation	– Speech. – Gesture (Bohn et al., 2020). – Stimuli as a whole (Vogt and Kauschke, 2017). – Unrelated aspect (Kelly et al., 2010a).
Type of iconic gesture	– Action (Stanfield et al., 2013). – Physical attributes (Dick et al., 2009). – Position (Beattie and Shovelton, 2002). – Typical vs. Atypical (Dargue and Sweller, 2018b).
Gesture-speech relationship	– Redundant (Holler et al., 2009) or Complementary (Kelly et al., 2004). – Congruent vs. Incongruent (Wu and Coulson, 2005). – Presence vs. Absence (Iani and Bucciarelli, 2017).
Type of stimuli	– Video clips (speech + gesture) (Kelly et al., 2010b). – Soundless video clips (Novack et al., 2016) – Live gestures (Kartalkanat and Göksun, 2020). – Target words/pictures (Bernardis et al., 2008; Wray et al., 2016). – Cartoons (Wu and Coulson, 2005).
Stimuli content	– Sentences (Momsen et al., 2020). – Single words (Sekine et al., 2020). – Narration (Macoun and Sweller, 2016). – Visual stimuli (Aussems and Kita, 2019).
Gesture length	– Full gesture (Kelly et al., 2007) or Stroke (So et al., 2013).
Origin of gesture	– Spontaneous (Holle et al., 2010) or Scripted (Holler et al., 2015).
Visibility of actor	– Knees up (Wolf et al., 2017). – Waist up (Dick et al., 2014). – Torso (Zhao et al., 2018). – Visible (Green et al., 2009) or Masked face (Zhao et al., 2018).

In conclusion, although iconic gestures convey useful information for the listener, the specificities of iconic gestural and linguistic information (such as the automaticity of integration, the relationship between gesture and speech, or the brain regions involved) open wide fields of possible research. On a theoretical level, qualifying iconic gestures as manual movements with a semantic relationship to the co-occurring speech does not allow for a complete understanding of the results presented in the various studies. There is a clear need for going further and systematically specifying the type of iconic gesture (action, shape, size, and position) used, its manner of presentation (video clips or face-to-face) and its relation to speech (redundant, complementary, and incongruent).

The automatic nature of gesture-speech integration remains an issue. However, the authors’ observations throughout this review support the theory of a modulated automaticity, depending on iconic gesture type, semantic overlap between gesture and co-occurring speech, particularly in the case of redundant vs. complementary, and individual differences in cognition. Although all electrophysiological studies have highlighted the existence of a semantic integration of information, they do not agree on the temporality of this integration (studies finding early and/or only late components). This disagreement mainly appears to stem from the use of different materials (simple vs. complex or redundant vs. complementary iconic gestures). Investigating early and late effects using similar material could help to resolve this issue.

Brain imaging studies have facilitated a deeper analysis, while showing the involvement of the left IFG, pSTS, and MTG in gesture-speech integration. The variations in results are consistent with the authors’ observation of the existence of different neural processing depending on the relationship between iconic gestures and speech. While existing studies have started to investigate the neural network involved in the processing of pantomimes, further research should explore the neural networks involved in the understanding of iconic gestures. As pointed out, brain imaging studies are particularly sensitive to the type of iconic gesture as well as the relationship it entertains with speech. Hence, future research could specify this information to make valid comparisons between studies as well as identifying the networks involved in different types of iconic gestures. Finally, since gesture-speech integration is a relatively recent field of investigation, studying the cognitive processes involved, such as working memory or attention, could allow for a better understanding of this integration.

## Author Contributions

KK: conceptualization and writing and editing – original draft preparation. IL and LL: supervision – reviewing and editing. WB: reviewing. MR: supervision – reviewing. All authors contributed to the article and approved the submitted version.

## Conflict of Interest

The authors declare that the research was conducted in the absence of any commercial or financial relationships that could be construed as a potential conflict of interest.
