# Supplementary material for: The Role of Iconic Gestures in Speech Comprehension: An Overview of Various Methodologies
Source: Front Psychol. 2021 Apr 29;12:634074. doi: 10.3389/fpsyg.2021.634074 (PMC8118122; doi:10.3389/fpsyg.2021.634074)
Supplement: Supplementary file 1 [file Table_1.DOCX]

| **Authors** | **Research type** | **Implicit vs Explicit** | **Tasks** | **Attention to** | **Iconic gesture type** | **Redund.**  **Vs**  **Compl.** | **Gesture/Speech relationship** | **Type of stimuli** | **Stimuli content** | **Bimodal vs Congruency** | **Gesture length** | **Origin of Gesture** | **Visibility of actor** |
| --- | --- | --- | --- | --- | --- | --- | --- | --- | --- | --- | --- | --- | --- |
| Aussems & Kita (2019) | Behavioral | Implicit | Obs.; Recognition task | Stimuli | Action | / | n/a | Soundless video clips + live gestures | Visual stimuli | Bimodal | Full gesture | Scripted | Body; Live |
| Beattie & Shovelton (1999) | Behavioral | Explicit | Quest. | Gesture | / | / | Gesture vs Speech vs Gesture + Speech | Video clips | Narration | / | Full gesture | / | / |
| Beattie & Shovelton (2001) | Behavioral | Explicit | Obs.; Free recall | Action; Object | Action; Phy. Att  C-VPT /  O-VPT | / | Gesture + Speech vs Speech | Video clips | Narration | Bimodal | Full gesture | Spontaneous | / |
| Bohn et al. (2020) | Behavioral | Explicit | Observation | Gesture | Action | / | Congruent vs Incongruent | Live soundless gestures (study 1) ; Soundless video clips (study 2) | Visual stimuli | Congruency | Full gesture | Scripted | Live (study 1); Waist up (study 2) |
| Botting et al. (2010) | Behavioral | Implicit | Obs. ; Picture relatedness | Stimuli | / | / | Sentence completion | Video clip + Target picture | Sentences | / | / | / | / |
| Cocks et al. (2011) | Behavioral | Implicit | Obs.; Picture relatedness | Stimuli | Action | / | Gesture vs Speech vs Speech-Gesture | Video clips | Short sentence | Bimodal | / | / | Knees up (masked face) |
| Cocks et al. (2009) | Behavioral | Implicit | Obs.; Picture relatedness | Stimuli | Action | / | Speech vs Speech-Gesture vs Gesture | Video clips + target pictures | Sentences | Bimodal | / | / | Torso (masked face) |
| Cocks et al. (2018) | Behavioral | Implicit | Obs.; Picture relatedness | Stimuli | Action | / | Speech + Gesture vs Speech vs Gesture | Video clip + Target picture | Sentences | Bimodal | / | / | Face masked |
| Dahl et al. (204) | Behavioral | Implicit | Cartoon image task; Quest. | Stimuli | / | / | Speech + Gesture vs Speech | Video clip | Narration | Bimodal | / | Spontaneous | Waist up |

Table I - Methodological variations in studies investigating gesture/speech integration

| **Authors** | **Research type** | **Implicit vs Explicit** | **Tasks** | **Attention to** | **Iconic gesture type** | **Redund.**  **Vs**  **Compl.** | **Gesture/Speech relationship** | **Type of stimuli** | **Stimuli content** | **Bimodal vs Congruency** | **Gesture length** | **Origin of Gesture** | **Visibility of actor** |
| --- | --- | --- | --- | --- | --- | --- | --- | --- | --- | --- | --- | --- | --- |
| Dargue & Sweller (2018) | Behavioral | Implicit | Obs., ; Recall ; Quest. | Stimuli | / | Redund. | Speech vs Congruent vs Incongruent | Video clips | Narration | Bimodal + Congruency | / | / | / |
| Dargue & Sweller (2018) | Behavioral | Implicit | Obs. (study 1);  Obs. ; Free recall (study 2) | Stimuli | n/a (study 1); Typical vs Atypical (study 2) | / | n/a (study 1); Speech-Gesture vs Speech (study 2) | Cartoon (study 1); Video clip (study 2) | Narration | Bimodal | / | Scripted | / |
| Dargue & Sweller (2020) | Behavioral | Implicit | Dual task paradigm; Free recall; Quest. | Stimuli | Typical ; Atypical | / | Speech vs Speech-Gesture | Video clips | Narration | Bimodal | / | Scripted | / |
| Demir et al. (2014) | Behavioral | Implicit | Obs.; Free recall | Stimuli | / | / | Speech vs Speech-Gesture vs Audio vs Cartoon | Video clips + soundless cartoon | Narration | Bimodal | / | Spontaneous | Knees up |
| Dimitrova et al. (2017) | Behavioral | Implicit | Obs.; Picture relatedness | Stimuli | / | Compl. Vs  Redund. | Speech + Gesture vs Speech vs Gesture | Live gesture + Picture target | Word | Bimodal | Full gesture | / | Live |
| Drijvers & Özyürek (2016) | Behavioral | Implicit | Obs.; Free recall | Speech | Action | / | Speech + Gesture vs Speech | Video clip + degraded sound | Single word | Bimodal | Full gesture | Spontaneous | Knees up |
| Drijvers & Özyürek (2020) | Behavioral | Implicit | Obs.; Free recall | Speech | Action | / | Speech vs Speech-Gesture | Video clips + degraded sound | Single word | Bimodal | Full gesture | Spontaneous | Knees up |
| Ferro et al. (1980) | Behavioral | Explicit | Gesture recognition task | Gesture | Pantomime | / | n/a | Live gesture | n/a | n/a | Full gesture | Scripted | Live |
| Gainotti & Lemmo (1976) | Behavioral | Explicit | Picture relatedness | Gesture | n/a | / |  | Soundless live + Picture target | Visual stimuli | / | Full gesture | Scripted | Live |
| Glasser et al. (2018) | Behavioral | Explicit | Obs.; Clip relatedness | Gesture | Action; Path | / | n/a | Video clips + animated clips | Sentences | Congruency | / | Spontaneous | Waist up |

Table I (continued) - Methodological variations in studies investigating gesture/speech integration

| **Authors** | **Research type** | **Implicit vs Explicit** | **Tasks** | **Attention to** | **Iconic gesture type** | **Redund.**  **Vs**  **Compl.** | **Gesture/Speech relationship** | **Type of stimuli** | **Stimuli content** | **Bimodal vs Congruency** | **Gesture length** | **Origin of Gesture** | **Visibility of actor** |
| --- | --- | --- | --- | --- | --- | --- | --- | --- | --- | --- | --- | --- | --- |
| Hadar & Pinchas-Zamir (2004) | Behavioral | Explicit | Matching word to gesture | Gesture | / | / | Gesture vs Gesture + text vs Gesture + text + speech | Video clips | Sentences | Bimodal | / | / | Waist up |
| Holler et al. (2009) | Behavioral | Implicit | Obs. | Stimuli | Size; Position | / | Speech + Gesture vs Speech vs Gesture | Live gesture + video clips | Narration | Bimodal | Full gesture | Scripted | Live; Waist up |
| Iani & Bucciarelli (2017) | Behavioral | Implicit | Obs. (study 1); Hand/Arm movements (study 2,3); Leg/feet movements (study 4);  Recall (study 1-4) | Stimuli | Action | / | Speech-Gesture vs Speech | Video clips | Sentences | Bimodal | / | / | Knees up |
| Kandana Arachchige et al. (2018) | Behavioral | Implicit | Stroop-like task | Unrelated aspect | Action; Phy. Att. | / | Congruent vs Incongruent | Video clips | Single word | Congruency | Full gesture | Scripted | Torso |
| Kartalkanat & Göksun (2020) | Behavioral | Implicit | Obs.; Free recall; Recognition task | Stimuli | Action, Path | / | Speech vs Speech-Gesture vs Speech-Movement | Live gesture | Narration | Bimodal | Full gesture | / | Live |
| Kelly et al. (2010b) | Behavioral | Explicit | Target relatedness | Gesture and/or Speech | Action | Redund. | Congruent vs Incongruent | Video clips (prime/target) | Single word | Congruency | / | Spontaneous | Torso |
| Macoun & Sweller (2016) | Behavioral | Implicit | Obs.; Free recall ; Quest. | Stimuli | Action; Phy. Att | Redund. vs Non Redund. | Speech vs Speech-Gesture vs Speech-Movements | Video clips | Narration | Bimodal | / | / | Waist up |
| Margiotoudi et al. (2014) | Behavioral | Explicit | Discrimination task | Speech & Gesture | Action | / | Congruent vs Incongruent | Video clips | Words | Congruency | / | / | / |
| Myiake & Sugimura (2018) | Behavioral | Implicit | Obs.; Picture relatedness | Stimuli | Action | / | Gesture vs Speech vs Speech-Gesture | Video clips | Sentences | Bimodal | Full gesture | / | Waist up |

Table I (continued) - Methodological variations in studies investigating gesture/speech integration

| **Authors** | **Research type** | **Implicit vs Explicit** | **Tasks** | **Attention to** | **Iconic gesture type** | **Redund.**  **Vs**  **Compl.** | **Gesture/Speech relationship** | **Type of stimuli** | **Stimuli content** | **Bimodal vs Congruency** | **Gesture length** | **Origin of Gesture** | **Visibility of actor** |
| --- | --- | --- | --- | --- | --- | --- | --- | --- | --- | --- | --- | --- | --- |
| Nagels et al. (2019) | Behavioral | Explicit | Word relatedness | Gesture & Speech | / | / | Congruent vs Incongruent | Video clips | Sentences | Congruency | / | / | / |
| Novack et al. (2016) | Behavioral | Implicit | Obs. | Stimuli | Action | / | n/a | Soundless video clips | / | Bimodal | Full gesture | Scripted | Torso |
| Özer & Göksun (2020) | Behavioral | Explicit | Matching target to prime | Gesture & Speech | Action | / | Congruent vs Incongruent | Action prime + video and word target | Single word | Congruency | / | Scripted | Torso |
| Perrault et al. (2019) | Behavioral | Explicit | Obs. ; Quest. | Gesture | Action | / | n/a | Soundless video clips | Visual stimuli | n/a | / | Scripted | Waist up |
| Ping et al. (2014) | Behavioral | Implicit | Picture relatedness (study 1); Arm or Leg movements (study 2) | Speech | / | / | Congruent vs Incongruent | Video clips + target pictures | Sentences | Bimodal + Congruency | / | / | Waist up |
| Quandt et al. (2012) | Behavioral | Explicit | Reach and grasp | Gesture | Action | / | / | Video clip | / | / | Full gesture | Scripted | Torso |
| Sekine et al. (2015) | Behavioral | Implicit | Obs.; Picture relatedness | Stimuli | Action | Compl. | Speech vs Gesture vs Speech-Gesture | Video clips (study 1); Live gesture (study 2) | Sentences | Bimodal | Full gesture | / | Torso (masked face) (study 1); Live (study 2) |
| So et al. (2012) | Behavioral | Implicit | Obs.; Free recall | Stimuli | Action | / | Speech-Gesture vs Speech-Movement vs Speech | Video clips | Single word | Bimodal | / | / | Waist up |
| So et al. (2013) | Behavioral | Implicit | Lexical decision task | n/a | Action; Phy. Att | / | Gesture vs Speech vs Speech-Gesture | Video clip + lexical target | Single word | Bimodal + Congruency | Stroke | / | Waist up |

Table I (continued) - Methodological variations in studies investigating gesture/speech integration

| **Authors** | **Research type** | **Implicit vs Explicit** | **Tasks** | **Attention to** | **Iconic gesture type** | **Redund.**  **Vs**  **Compl.** | **Gesture/Speech relationship** | **Type of stimuli** | **Stimuli content** | **Bimodal vs Congruency** | **Gesture length** | **Origin of Gesture** | **Visibility of actor** |
| --- | --- | --- | --- | --- | --- | --- | --- | --- | --- | --- | --- | --- | --- |
| Stanfield et al. (2013) | Behavioral | / | Obs.; Picture relatedness | Stimuli | Action | Compl. | Congruent vs Incongruent | Live gestures + pictures | Sentences | Congruency | Full gesture | / | Live |
| Van Wermeskerken et al. (2016) | Behavioral | Implicit | Obs.; Route, Location, Street retention | Stimuli | Depictive | / | Speech vs Speech-Gesture vs Speech-Tracing vs Speech-Tracing-Gesture | Video clips | Narration | Bimodal | / | / | Waist up |
| Vogt & Kauschke (2017) | Behavioral | Implicit | Obs.; Learning; Naming ; Comprehension | Stimuli | Action; Phy. Att | / | Speech-Gesture vs Speech-movement | Live gesture | Sentences | Bimodal | / | / | Waist up |
| Wray et al. (2016) | Behavioral | Implicit | Obs.; Picture relatedness | Stimuli |  | / | Sentence completion | Video clip + Target picture | Sentences | / | / | / | / |
| Wu & Coulson (2014) | Behavioral | Implicit | Picture relatedness task (study 1) ; Dual task paradigm (studies 2-4) | Speech | Action; Phy. Att. | / | Congruent vs Incongruent | Title + discourse prime + picture probe | Words | Congruency | / | Spontaneous | Knees up (masked face) |
| Yap et al. (2011) | Behavioral | Implicit | Lexical decision task | n/a | Action; Phy. Att | / | Gesture + Speech vs Speech | Soundless video clips + lexical target | Visual stimuli | / | Full gesture (study 1); Stroke (study 2) | / | / |
| Bernardis et al.(2008) | Behavioral; EEG | Implicit | Word reading | Stimuli | n/a (pantomime) | / | n/a | Soundless video clip prime + target word | Visual stimuli | Congruency | Full gesture | / | Torso (masked face) |
| Drijvers & Özyürek (2018) | Behavioral; EEG | Implicit | Obs.; Cued-verb recall task | Speech | Action | / | Congruent vs Incongruent | Video clips + degraded sound | Single word | Congruency | Full gesture | / | Knees up |
| He et al. (2018) | Behavioral; EEG | Implicit | Content judgment task | Speech | Pantomime | / | Speech vs Speech-Gesture | Video clips | Sentences | Bimodal | / | / | Knees up |

Table I (continued) - Methodological variations in studies investigating gesture/speech integration

| **Authors** | **Research type** | **Implicit vs Explicit** | **Tasks** | **Attention to** | **Iconic gesture type** | **Redund.**  **Vs**  **Compl.** | **Gesture/Speech relationship** | **Type of stimuli** | **Stimuli content** | **Bimodal vs Congruency** | **Gesture length** | **Origin of Gesture** | **Visibility of actor** |
| --- | --- | --- | --- | --- | --- | --- | --- | --- | --- | --- | --- | --- | --- |
| Holle & Gunter (2007) | Behavioral; EEG | Explicit (study 1); Implicit (study 2) | Gesture/Speech compatibility (study 1); Obs.(study 2) | Gesture (study 1); Stimuli (study 2) | Action; Phy. Att. | / | Dominant vs Subordinate | Video Clips | Sentences | Congruency | Full gesture | Spontaneous | Waist up (masked face) |
| Kelly et al. (2007) | Behavioral; EEG | Implicit | Speech recognition | Speech | Phy. Att. | / | Congruent vs Incongruent; Intent vs Non intent | Video clips | Single word | Congruency | Full gesture | / | Torso |
| Kelly et al. (2009) | Behavioral; EEG | Implicit | Obs.; Learning; Memory task | Stimuli | Action | / | Congruent vs Incongruent vs Absence of gesture vs Repeated Speech | Live gestures (study 1); Video clips (study 2) | Single word | Bimodal + Congruency | Full gesture | / | Live (study 1); Waist up (study 2) |
| Kelly et al. (2010a) | Behavioral; EEG | Implicit | Stroop-like task | Unrelated aspect | Action | Redund. | Congruent vs Incongruent | Video clips | Single word | Congruency | Stroke | / | Torso |
| Momsen et al. (2020) | Behavioral; EEG | Implicit | Dual task paradigm; Observation | Stimuli | Action; Phy. Att | / | Congruent vs Incongruent | Video clips + picture probe | Sentences | Congruency | Stroke | / | Torso (masked face) |
| Sekine et al. (2020) | EEG; Behavioral | Implicit | Obs.; Recognition task | Stimuli | Action | / | Congruent vs Incongruent | Video clips | Single word | Congruency | Full gesture | Spontaneous | Knees up |
| Wu & Coulson (2005) | EEG; Behavioral | Explicit (study 1); Implicit (study 2) | Matching gesture with cartoon (study 1); Matching word to probe (study 2) | Gesture (study 1); Speech (study 2) | Action; Phy. Att. | / | n/a | Soundless cartoon + soundless video clips + word probe (study 2) | Visual stimuli | Congruency | Stroke | Spontaneous | Waist up |
| Wu & Coulson (2007a) | Behavioral; EEG | Explicit (study 1); Implicit (study 2) | Word match (study 1);  Obs. (study 2) | Speech (study 1);  Stimuli (study 2) | Action; Phy. Att. | n/a | n/a | Soundless video clips + target words | Visual stimuli | Congruency | / | Spontaneous | Waist up |

Table I (continued) - Methodological variations in studies investigating gesture/speech integration

| **Authors** | **Research type** | **Implicit vs Explicit** | **Tasks** | **Attention to** | **Iconic gesture type** | **Redund.**  **Vs**  **Compl.** | **Gesture/Speech relationship** | **Type of stimuli** | **Stimuli content** | **Bimodal vs Congruency** | **Gesture length** | **Origin of Gesture** | **Visibility of actor** |
| --- | --- | --- | --- | --- | --- | --- | --- | --- | --- | --- | --- | --- | --- |
| Wu & Coulson (2007b) | EEG; Behavioral | Implicit | Attention task; word recognition | Stimuli | Action; Phy. Att. | Compl. | Congruent vs Incongruent | Video clips + picture probe + word | Sentences | Congruency | / | Spontaneous | Knees up |
| Beattie et al. (2010) | Eye track; Behavioral | Implicit | Content questions | Stimuli | High/Low Span;  C-VPT /  O-VPT | / | / | Video clips | Narration | n/a | Full gesture | Scripted | Knees up |
| Drijvers et al. (2019) | Eye track; Behavioral | Implicit | Obs.; Cued-recall | Speech | Action | / | Speech vs Speech-Gesture | Video clips + degraded sound | Single word | Bimodal | Full gesture | / | Knees up |
| Demir-Lira et al. 2018) | fMRI; Behavioral | Implicit | Obs.; Recognition task | Stimuli | Action | Compl. vs Redund. | Speech-Gesture vs Speech | Video clips | Narration | Bimodal | Full gesture | / | Waist up |
| Green et al. (2009) | fMRI; Behavioral | Implicit | Attentional task | Unrelated aspect | Action; Phy. Att | / | Gesture vs Speech | Video clips | Sentences | Bimodal | Full gesture | Spontaneous | Waist up |
| Holle et al. (2008) | fMRI; Behavioral | Implicit | Word relatedness | Stimuli | Action; Phy. Att. | / | Dominant vs Subordinate | Video clips + target word | Sentences | n/a | Full gesture | Spontaneous | Knees up (masked face) |
| Straube et al. (2011) | fMRI; Behavioral | Implicit | Attention task; Memory task | Novelty | Action; Phy. Att | / | Speech vs Gesture vs Speech-Gesture | Video clips | Sentences | Bimodal | Full gesture | Spontaneous | Knees up |
| Straube et al. (2018) | fMRI; Behavioral | Implicit | Content judgement task | Speech | Pantomime | / | Speech-Gesture vs Speech | Video clips | Sentences | Bimodal | Full gesture | Spontaneous | Knees up |
| Willems et al. (2009) | fMRI; Behavioral | Implicit | Recognition task | Stimuli | Action | / | Congruent vs Incongruent / Audio vs Gesture vs Speech-Gesture | Video clips | Sentences | Bimodal + Congruency | / | / | Torso |

Table I (continued) - Methodological variations in studies investigating gesture/speech integration

| **Authors** | **Research type** | **Implicit vs Explicit** | **Tasks** | **Attention to** | **Iconic gesture type** | **Redund.**  **Vs**  **Compl.** | **Gesture/Speech relationship** | **Type of stimuli** | **Stimuli content** | **Bimodal vs Congruency** | **Gesture length** | **Origin of Gesture** | **Visibility of actor** |
| --- | --- | --- | --- | --- | --- | --- | --- | --- | --- | --- | --- | --- | --- |
| Wolf et al. (2017) | fMRI; Behavioral | Explicit; Implicit | Conventionality vs hand movement detection | Gesture | / | / | / | Video clips | Narration | n/a | Full gesture | Spontaneous | Knees up |
| Cohen-Maximov et al. (2015) | tDCS; Behavioral | Explicit | Semantic decision; Flanker task | Gesture | n/a  (pantomime) | / | Congruent vs Incongruent | Video clip prime + target word | Single word | Congruency | / | / | Torso (masked face) |
| Zhao et al. (2018) | TMS; Behavioral | Implicit | Stroop-like task | Unrelated aspect | Action | / | Congruent vs Incongruent | Video clips | Single word | Congruency | Stroke | / | Torso |
| Eggenberger et al. (2016) | Behavioral | Explicit | Congruency gesture/speech | Gesture; Speech | n/a | / | Congruent vs Incongruent | Video clip | Sentences | Congruency | / | / | Waist up |
| Schulke & Straube (2019) | Behavioral; tDCS | Explicit | Congruency gesture/speech | Gesture; Speech | Action; Phy. Att |  | Related vs Unrelated | Video clip | Sentences | Congruency | / | / | Knees up |
| Drijvers et al. (2018) | MEG; Behavioral | Implicit | Obs.; Cued-recall task | Speech | Action | Compl. | Speech-Gesture vs Speech | Video clips + degraded sound | Single word | Bimodal | Full gesture | Spontaneous | Knees up |
| Habets et al. (2011) | EEG | Implicit | Obs. | Stimuli | Action | / | Congruent vs Incongruent | Video clips | Single word | Congruency | Stroke | Scripted | Knees up (no face) |
| Kelly et al. (2004) | EEG | Implicit | Speech recognition | Speech | Phy. Att. | Redund. Vs Compl. | Congruent vs Incongruent vs Speech | Video clips | Words | Bimodal + Congruency | / | Scriped | Waist up |
| Obermeier & Gunter (2014) | EEG | Implicit | Obs. | Stimuli | / | / | Dominant vs Subordinate | Video clips | Sentences | Congruency | Full gesture | / | Knees up (face masked) |
| Özyürek et al. (2007) | EEG | Implicit | Observation | Stimuli | Action | / | Congruent vs Incongruent | Video clips | Sentences | Congruency | Stroke | Scripted | Knees up (masked face) |
| Dick et al. (2009) | fMRI | Implicit | Obs. | Stimuli | Phy. Att | / | Gesture vs Grooming vs Speech vs Audio | Video clips | Narration | Bimodal | Full gesture | Scripted | Waist up |

Table I (continued) - Methodological variations in studies investigating gesture/speech integration

| **Authors** | **Research type** | **Implicit vs Explicit** | **Tasks** | **Attention to** | **Iconic gesture type** | **Redund.**  **Vs**  **Compl.** | **Gesture/Speech relationship** | **Type of stimuli** | **Stimuli content** | **Bimodal vs Congruency** | **Gesture length** | **Origin of Gesture** | **Visibility of actor** |
| --- | --- | --- | --- | --- | --- | --- | --- | --- | --- | --- | --- | --- | --- |
| Dick et al. (2012) | fMRI | Implicit | Obs.; Quest. | Stimuli | / | / | n/a | Video clips | Sentences | n/a | / | / | / |
| Dick et al. (2014) | fMRI | Implicit | Obs. ; Quest. | Stimuli | / | Redund. Vs Compl. | Congruent vs Incongruent vs Speech | Video clips | Sentences | Bimodal | Full gesture | Rehearsed | Waist up |
| Holle et al. (2010) | fMRI | Implicit | Obs. | Stimuli | Action | / | Speech vs Gesture vs Speech-Gesture | Video clips + degraded sound | Sentences | Bimodal | Full gesture | Spontaneous | Knees up (masked face) |
| Holler et al. (2015) | fMRI | Implicit | Attention task | Stimuli | Action | / | Speech vs Speech-Gesture | Video clips | Sentences | Bimodal | / | Scripted | Knees up |
| Willems et al. (2007) | fMRI | Implicit | / | Stimuli | Action; Phy. Att. | Redund. | Congruent vs Incongruent | Video clips | Sentences | Congruency | Stroke | Scripted | Waist up (masked face) |

Table I (continued) - Methodological variations in studies investigating gesture/speech integration

List of abbreviations: Obs. = Observation; Quest. = Questionnaire; Phy. Att. = Physical Attribute; Redund. = Redundant; Compl. = Complementary; Intent. = Intentionality
